# Hyperendemicity associated with increased dengue burden

**DOI:** 10.1098/rsif.2021.0565

**Published:** 2021-09-15

**Authors:** Jue Tao Lim, Borame Sue Dickens, Ken Wei Tan, Joel Ruihan Koo, Annabel Seah, Soon Hoe Ho, Janet Ong, Jayanthi Rajarethinam, Stacy Soh, Alex R. Cook, Lee Ching Ng

**Affiliations:** ^1^ Environmental Health Institute, National Environmental Agency, Singapore; ^2^ Saw Swee Hock School of Public Health, National University of Singapore and National University Health System, Singapore; ^3^ School of Biological Sciences, Nanyang Technological University, Singapore

**Keywords:** dengue, modelling, Bayesian statistics, hyperendemicity, serotypes

## Abstract

Over 105 million dengue infections are estimated to occur annually. Understanding the disease dynamics of dengue is often difficult due to multiple strains circulating within a population. Interactions between dengue serotype dynamics may result in complex cross-immunity dynamics at the population level and create difficulties in terms of formulating intervention strategies for the disease. In this study, a nationally representative 16-year time series with over 43 000 serotyped dengue infections was used to infer the long-run effects of between and within strain interactions and their impacts on past outbreaks. We used a novel identification strategy incorporating sign-identified Bayesian vector autoregressions, using structural impulse responses, historical decompositions and counterfactual analysis to conduct inference on dengue dynamics post-estimation. We found that on the population level: (i) across-serotype interactions on the population level were highly persistent, with a one time increase in any other serotype associated with long run decreases in the serotype of interest (range: 0.5–2.5 years) and (ii) over 38.7% of dengue cases of any serotype were associated with across-serotype interactions. The findings in this paper will substantially impact public health policy interventions with respect to dengue.

## 1. Introduction

Dengue is a multi-serotype pathogen responsible for over 105 million infections globally a year [1]. Serotype dynamics play a crucial role in the persistent burden of dengue worldwide. Immune response after infection with one serotype confers temporary cross-immunity against other serotypes of the same pathogen as evidenced from laboratory [2] and cohort studies [3], but in the longer term when cross-immunity wanes, secondary infection may be possibly more severe. While there is substantial empirical evidence of short-term cross-immunity, the duration of cross-immunity remains difficult to establish [4], and its corresponding impacts on dengue transmission at the population level are also less widely studied. In the case of dengue, secondary infection can lead to potentially life-threatening conditions, such as dengue hemorrhagic syndrome and/or dengue shock syndrome [5]. Owing to the hyperendemic nature of dengue in many regions, where multiple serotypes of dengue are in active circulation [1], sustained reinfection remains a constant threat to individuals. On the population level, temporary cross protection and the forward risk of secondary infections in hyperendemic regions result in complex temporal dynamics where it is difficult to infer the underlying epidemiological factors giving rise to dengue outbreaks [4].

Multi-strain dynamics are a posited cause of dengue outbreaks. Cyclical epidemics are observed to comprise alternating serotypes in many localities where dengue is hyperendemic [6]. There is evidence that switches between serotypes and/or the displacement of the predominant serotype may serve as warnings for looming outbreaks [6] and establishment of multiple genotypes over time causes selection of genotypes of higher fitness, which may increase the risk of outbreaks even under conditions where vector breeding potential is low [7]. Prior work has however suggested that waning cross immunity is a necessary condition for large outbreaks to occur in multi-annual cycles [8]. Therefore, it is crucial to account for multi-strain dynamics in order to understand the underlying epidemic process of dengue.

The increased worldwide burden of dengue, coupled with the importation and establishment of new serotypes has led to dengue hyperendemicity in an increasing number of localities [1]. On the population level, this makes the efficacy of recently developed vaccines difficult to establish, due to (i) the co-circulation of multiple serotypes and/or (ii) low sero-prevalence of dengue. As temporal changes in cross-protection create challenges in epidemiological studies, vaccine trials face substantial inferential headwinds that make it difficult to infer vaccine efficacy when deployed on the population level, especially if individuals were infected a varying number of times to a varying number of serotypes [4]. Varying seroprevalence rates especially among younger age groups also make the application of vaccines potentially problematic [5,9]. A detailed understanding of the transmission dynamics of dengue on the population level would thus be necessary, to formulate possible intervention strategies to stave the onward transmission of dengue virus.

For the past three decades in Singapore, dengue is hyperendemic with four serotypes in active circulation. Epidemics occur in multi-annual cycles, where increasingly larger surges in the number of dengue cases in 2012–2015, 2015–2016 and 2020 have been reported [10]. Prior studies have also attributed these outbreaks to the low sero-prevalence of dengue among younger age groups, due to successful vector control practices, as well as dengue hyperendemicity [11]. While virological studies have qualitatively described that these outbreaks are pre-empted by a switch in the pre-dominant serotype [6] or fitter genotypes [7], the explicit impacts of hyperendemicity on dengue burden have not been quantified directly.

There are a large number of dengue modelling studies under the setting of multiple co-circulating serotypes. These include multi-strain compartmental models which found that cross-immunity is necessary to generate persistent cycles of dengue epidemics [8], as well as other generalizations to allow for time varying transmission potential and incorporation of vital dynamics [12]. One advantage of compartmental models is having a convenient interpretation of the underlying biological process. However, calibrating these models may be cumbersome for multi-strain, vector borne diseases such as dengue—requiring many assumptions such as the renewal rate of the disease vector and seasonality.

The vector autoregression (VAR) approach, where the dynamics for multiple time series can be taken into account within the regression structure [13] is one potential solution for inferring underlying multi-strain disease processes. It requires less explicit assumptions of the underlying disease process and is computationally straightforward to estimate the underlying parameters of interest. Past work has employed this technique to infer underlying ecological processes [14] as well as the spatio-temporal spread of diseases [15], but no work has attempted to employ this class of models for inferring hyperendemic disease dynamics on the population level.

In this study, we investigate the dynamic signature of dengue in dengue hyperendemic Singapore as our key study example. In particular, we wish to understand how interactions within and across dengue serotypes on the population level are responsible for transmission persistence and elevated disease burden. Weekly case counts consisting of serotype-specific, laboratory confirmed dengue illness from 2006 to 2020 in Singapore were analysed, using a novel inference strategy incorporating Bayesian VARs (BVARs) and sign restrictions. We propose three approaches using the identified VAR model to understand dengue dynamics across multiple serotypes, namely through sign-restricted structural impulse responses, historical decompositions and counterfactual analysis of past outbreaks.

## 2. Method

### 2.1. Data requirements

The circulating DENV populations were monitored through a virus surveillance programme jointly conducted by the Ministry of Health, Singapore and National Environmental Agency, Singapore. Blood samples from suspected dengue patients who sought treatment at general practitioners, public/private hospitals and polyclinics were tested for the evidence of DENV by using either NS1 antigen or polymerase chain reaction (PCR) assays. Serotype and genotype analyses were performed on a weekly basis. DENV-positive sera were further analysed to determine the serotype of DENV by using a real time reverse-transcription PCR (RT-PCR) assay [16]. At Environmental Health Institute, one of the public health laboratories in Singapore, all DENV-positive sera that failed serotype screening by the real time RT-PCR assay were subjected to a modified semi-nested conventional PCR assay [16]. For our analysis, we used weekly dengue case counts consisting of serotype-specific, laboratory confirmed dengue illness for 14 years, from epidemiological week 1 (EW-1) 2006 through EW-52 2020.

### 2.2. Identification strategy

In summary, our approach to identifying hyperendemic disease dynamics is through first estimating the reduced-form BVAR and then recovering the BVAR structural parameters through biologically motivated sign restrictions [17].

The dynamics of dengue were considered in the following 4-variate reduced-form VAR(p) model, where p refers to the maximum number of autoregressive lags. The 4-variate form corresponds to the four serotypes which are in active circulation in Singapore:

2.1 $$y_{t}=v+A_{1}y_{t-1}+A_{2}y_{t-2}+\cdots +A_{p}y_{t-p}+u_{t},$$

for *t* = 1, …, *T*, where $u_{t}\overset{\mathrm{iid}}{∼}N(0_{4\times 1},\Sigma_{u})$. Here *y*_*t*_ is the contemporaneous vector of 4 × 1 time series, *v*_4×1_ the intercept term, *A*_*i*,4×1_ the autoregressive terms. *u*_*t*_ is the zero mean normally distributed error term with variance covariance matrix $\Sigma_{u,4\times 4}$. Estimated parameters consist of the autoregressive terms and the variance–covariance matrix, which was conducted through a direct sampling Markov chain Monte Carlo approach as described explicitly in electronic supplementary material, appendix A.

However, the variance covariance matrix $\Sigma_{u,4\times 4}$ consists of contemporaneously correlated errors which cannot provide clear interpretations of how one serotype independently affects the others across time on the population level. The primary goal of this analysis is thus recovering the structural BVAR, with mutually uncorrelated errors as follows:

2.2 $$B_{0}y_{t}=B_{1}y_{t-1}+\cdots +B_{p}y_{t-p}+w_{t},$$

where *B*_*i*_ is the structural autoregressive matrix of dimension 4 × 4. $w_{t}\overset{\mathrm{iid}}{∼}(0_{4\times 1},\Sigma_{w})$ denotes the white noise vector of dimension 4 × 1. The framework is structural as elements of *w*_*t*_ are mutually uncorrelated and have clear interpretations in terms of underlying multi-strain disease dynamics. The model is expressed in reduced form by left multiplying (2.2) by $B_{0}^{-1}$ such that

$$\begin{array}{rl} y_{t} & =B_{0}^{-1}B_{1}y_{t-1}+\cdots +B_{0}^{-1}B_{p}y_{t-p}+B_{0}^{-1}w_{t}\\ & =A_{1}y_{t-1}+\cdots +A_{p}y_{t-p}+u_{t}. \end{array}$$

Recovering the structural representation thus requires knowledge of *B*_0_ or $B_{0}^{-1}$, the matrix expressing contemporaneous relationships among the model’s endogenous variables. We have thus adapted the sign restriction identification strategy of [18] to search for candidate solutions for *B*_0_, so that inference can be conducted. The sign restrictions used in the paper were biologically motivated from prior work on clinical studies [2] and population level dengue dynamics [4], where there is substantial evidence that infected individuals are known to be ascribed short-term cross immunity to other serotypes of dengue. These sign restrictions were allowed to hold for 100 weeks, corresponding to the median duration of prior estimates of cross-immunity in individuals [2,4]. In terms of sign restrictions, this implies that a positive increase in case counts (+) for a specific serotype would lead to contemporaneous negative (−) decreases in all other serotypes in the structural impact multiplier matrix:

2.3 $$u_{t}\equiv \left(\begin{array}{c} u_{\mathrm{denv}-1}\\ u_{\mathrm{denv}-2}\\ u_{\mathrm{denv}-3}\\ u_{\mathrm{denv}-4} \end{array}\right)\equiv \left[\begin{array}{cccc} + & - & - & -\\ - & + & - & -\\ - & - & + & -\\ - & - & - & + \end{array}\right]\left(\begin{array}{c} w_{\mathrm{denv}-1}\\ w_{\mathrm{denv}-2}\\ w_{\mathrm{denv}-3}\\ w_{\mathrm{denv}-4} \end{array}\right)\equiv B_{0}^{-1}w_{t}.$$

To elaborate, a positive increase in serotype 1 (*w*_denv−1_, +) is known and will lead to negative responses on serotypes 2 (*w*_denv−2_, −), 3 (*w*_denv−3_, −) and 4 (*w*_denv−4_, −). An efficient search strategy, which nests estimation of both reduced form and structural BVAR parameters is explicitly delineated in electronic supplementary material, appendix A.

### 2.3. Inference strategy

We used structural impulse responses and historical decompositions as inference strategies for the BVAR. The structural impulse response is the response of each serotype to a one-time impulse in the structural errors. A unit shock on some serotype and the subsequent impulse response value on other serotypes can be thought of as the corresponding impact of an independent increase in the case counts of a shocked serotype on case counts of the other serotypes over time. This shock could be taken as a rise in case counts for a particular serotype, induced by exogenous factors, such as favourable climate conditions and increases in vector population. Whereas the historical decomposition further quantifies how much a given structural shock explains the historically observed fluctuations in the VAR variables. That is, know the cumulative effect of a given structural shock on each variable at every given point in time. In the case of dengue, this gives us valuable information on how prior large increases or decreases in a particular serotype contribute to historical outbreaks.

Historical counterfactuals can also be computed given the historical decomposition, to indicate how each serotype of interest would have evolved, had one replaced all realizations of shock *j* by zero, while preserving the remaining structural shocks in the model:

$$y_{kt}-{\hat{y}}_{\;jk,t}.$$

If the counterfactual exceeds *y*_*kt*_, this means that structural shock to variable *j* lowers *y*_*kt*_. A counterfactual below the observed *y*_*kt*_ means that the shock of interest raised *y*_*kt*_. The difference between the actual *y*_*kt*_ and the counterfactual tells us how much shock *j* affected *y*_*kt*_ at time *t*. Following Uhlig [18], we report our results using 68% credible intervals.

### 2.4. Model assessment

Gewecke convergence diagnostic tests [19] and visual assessment of trace plots [20] were used to determine convergence of Markov chain Monte Carlo procedures. The number of lags for our model was selected post-estimation, by sequentially increasing the number of lags for our model specification and visual inspection of residual autocorrelation in all time series after [21]. Model diagnostic summary statistics include the mean-square error, *R*^2^ and Bayes factor, to determine model fit and evidence [20]. Quantile–quantile plots and posterior predictive checks were used to determine whether the proposed BVAR model characterized the distribution of data across serotypes appropriately [13]. Results for model assessment are reported in electronic supplementary material, appendix A.

## 3. Results

### 3.1. Persistence in within and across serotype interactions on the population level

Impulse responses as computed from the model can be interpreted as the independent, population level effect of a hypothetical increase/decrease in any serotype on every serotype accounted for within the model. Our analysis using impulse responses indicate that in general, for an independent increase in any serotype of interest within a population, there were long run increases on the serotype of interest and long run decreases on all other serotypes within a population. These magnitudes vary across serotypes as we will elaborate below.

**Figure 1.**
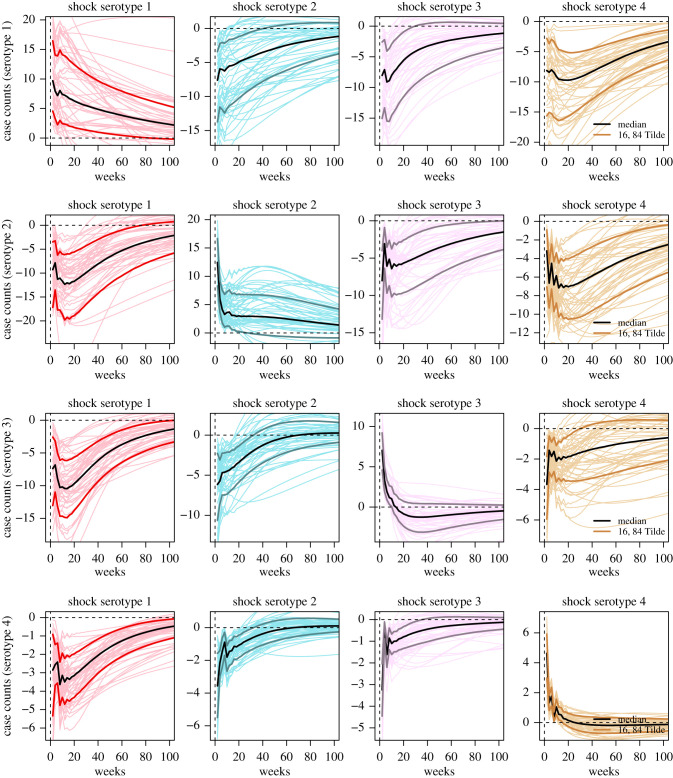
Impulse response functions for each serotype given a standard deviation shock in another. Dark solid coloured lines represent the posterior median impulse response for 0–100 weeks after an initial shock in another serotype. Lighter coloured lines represent the 16th and 84th posterior quantile impulse response for 0–100 weeks after an initial shock in another serotype. Fine lines represent posterior impulse response draws for 0–100 weeks after an initial shock in another serotype.

A hypothetical increase in DENV-1 within the population took the longest to reduce to zero. This was indicated by 68% credible interval values for the impulse response of DENV-1 on itself which did not approach 0 even after 50 weeks from the initial increase. However, for DENV-2, DENV-3 and DENV-4, the 68% credible intervals for impulse responses all approached 0 before 30 weeks. When looking at the increases applied to each serotype on itself, there was a tendency for increases in DENV-1, DENV-2, DENV-3 and DENV-4 to persist the longest to shortest, respectively (figure 1).

Impulse responses also provide the independent effect of an increase in the serotype of interest on another serotype. Our results indicate that a positive increase in DENV-1 and DENV-4 created the longest run negative impact on all other serotypes. An increase in DENV-1 led to long run decreases on all other serotypes. In particular, these decreases had 68% credible intervals which lasted for 60, 65 and more than 100 weeks for DENV-4, DENV-3 and DENV-2 respectively. For a positive increase in DENV-4, decreases on all other serotypes have 68% credible intervals approaching zero only at greater than 100 weeks, except for DENV-3, where 68% credible intervals approached zero at around 20 weeks from the initial increase (figure 1).

Notably, the independent effect of an increase in DENV-3 which had led to a corresponding decrease in DENV-2 was also relatively persistent, with 68% credible intervals approaching 0 at only more than 100 weeks. In comparison, the independent effect of an increase in DENV-3 on DENV-1 and -4 is less persistent, with 68% credible intervals approaching 0 before 40 weeks. Lastly, the independent effect of an increase in DENV-2 had the shortest corresponding decreases in all other serotypes, with decreases on all other serotypes having 68% credible intervals which approached zero before greater than 40 weeks (figure 1).

### 3.2. Outbreak size attributable to hyperendemicity

**Figure 2.**
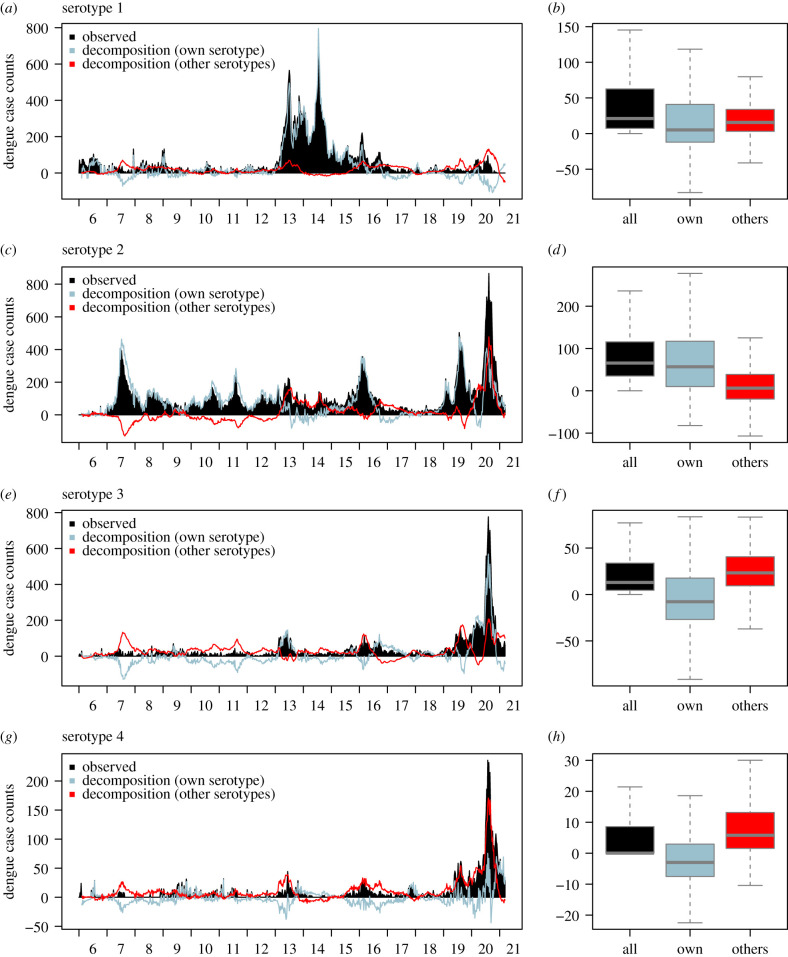
(*a*) Time series plot of DENV-1 case counts with historical decompositions of DENV-1 and all other serotypes. (*b*) Summary of weekly case contribution from DENV-1 and all other serotypes from historical decompositions. (*c*) Time series plot of DENV-2 case counts with historical decompositions of DENV-2 and all other serotypes. (*d*) Summary of weekly case contribution from DENV-2 and all other serotypes from historical decompositions. (*e*) Time series plot of DENV-3 case counts with historical decompositions of DENV-3 and all other serotypes. (*f*) Summary of weekly case contribution from DENV-3 and all other serotypes from historical decompositions. (*g*) Time series plot of DENV-4 case counts with historical decompositions of DENV-4 and all other serotypes. (*h*) Summary of weekly case contribution from DENV-4 and all other serotypes from historical decompositions.

Historical decompositions allow us to delineate how past outbreaks were the result of all past within- and across-serotype interactions on the population level. From 2006 to 2020, there were three notable outbreaks: a large, sustained 2-year DENV-1 outbreak in 2013–2014, where weekly case counts surpassed their observed historical number at 891, a DENV-2 outbreak in 2015–2016, where weekly reported DENV-2 case counts gradually increased to over 350 and a DENV-2/3/4 outbreak in 2020. Notably, in these periods, there had also been an elevated number of DENV-3 case counts (figure 2).

**Figure 3.**
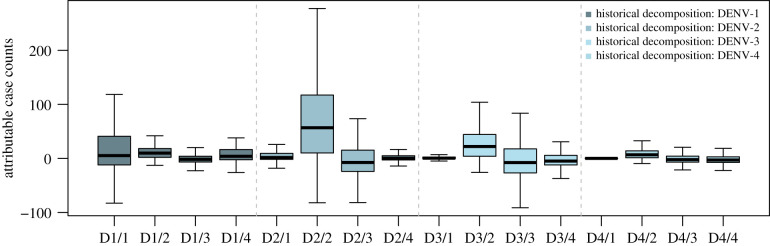
Summary boxplot for historical decompositions within and between serotypes. Boxplots from left to right represent: the historical contributions of DENV-1 to DENV-1,2,3,4, DENV-2 to DENV-1,2,3,4, DENV-3 to DENV-1,2,3,4 and DENV-4 to DENV-1,2,3,4.

Using historical decomposition analysis for the DENV-1 2012–2015 outbreak, for the period of epidemiological week (EW) 1, 2013 to EW 52, 2014, a total of 267, 71 and 704 DENV-1 case counts can be attributed to past changes in DENV-2, 3 and 4, respectively, which correspond to 0.95%, 1.33% and 2.5% of DENV-1 case counts in that same period. Counterfactually removing all other serotypes would have approximately reduced the size of the DENV-1 outbreak by 4.8%, by an absolute number of 1343 reported DENV-1 cases. Whereas for the DENV-2 2015–2016 outbreak, for the period of EW 1, 2015 to EW 52, 2016, a total of 2179, 1032 and −330 DENV-2 case counts can be attributed to past changes in DENV-1, 3 and 4 respectively, which correspond to 18.7%, 8.8% and –2.8% of case counts in that same period. Counterfactually removing all other serotypes would have approximately reduced the size of the DENV-2 epidemic by 24.7%, by an absolute number of 2881 reported DENV-2 cases. Lastly, for the DENV-2/3/4 outbreak for the period of EW 1, 2020 to EW 52, 2020, we found that more than 57.8%, 26.8% and 81.4% of DENV-2/3/4 cases can be attributed to past changes in all other serotypes respectively, corresponding to 9478, 3543 and 3452 additional DENV-2/3/4 cases in this period (figures 2 and 3).

The severity of dengue outbreaks is most attributed to changes in other serotypes (with respect to the serotype of interest) in the DENV-2/3/4 outbreak of 2020, followed by DENV-2 outbreak in 2015–2016 and the DENV-1 outbreak of 2013–2014—where a majority of cases are linked to past changes in DENV-1 itself. In terms of the overall impacts of across-serotype interactions, historical decompositions indicate that over 38.7% of reported case counts of all serotypes are attributed to contributions from another serotype. In particular, 64%, 81.7%, 17.9% and −10.6% of DENV-1,2,3,4 case counts were due to past patterns in their respective serotype and around 36.0%, 18.3%, 82.1% and 110.6% of DENV-1,2,3,4 case counts were attributed to past patterns across other serotypes. We found that the historically predominant DENV-2 contributed the most to case counts of DENV-1,3,4, at 20.5%, 85.2% and 90.7%, respectively, from 2006 to early 2021 (figures 2 and 3).

## 4. Discussion

A large body of work has been published on the potential causes for persistent dengue transmission. Some of these include population renewal [22] and spatio-temporal heterogeneity which may lead to source–sink dynamics on the national [23] and regional scale [15]. Vector dynamics and immunological interactions [4,24] are also posited as drivers of multi-annual cycles of outbreaks. Our analysis explicitly revealed the persistence of dengue transmission for each specific serotype, as well as the persistence of across serotype dynamics. An independent increase in the number of cases for every serotype was found to only reach zero in at least 10 weeks, with the greatest level of persistence found in DENV-1, lasting more than 60 weeks. These results demonstrate that dengue transmission is likely to continue over time, despite the impacts of interactions across serotypes on the population level.

The seminal studies by Sabin [2] and Reich [4] showed evidence of short-term cross-immunity to other serotypes of dengue, once an individual is infected with a specific serotype, but in the long term, secondary infections may lead to severe illness due to antibody dependent enhancement. Our analysis differs from previous work inferring how cross-immunity functions between individuals. Rather, we are concerned with how increases in the transmission of one serotype lead to changes in the transmission of other serotypes on the population level through impulse response analysis. Yet, parallels could be drawn on the duration of cross-immunity within an individual and the duration of across serotype interactions on the population level. Across-serotype interactions were estimated to be negatively persistent for up to 60 weeks, and cross-immunity in individuals was estimated to be from 6 months up to 3 years [2,4]. We note that the proposed sign-restriction framework preserves an agnostic view on the magnitude and duration of interactions between serotypes on the population level, although estimates from [2,4] were used to inform contemporaneous cross-immunity in the population (i.e. a rise in the number of dengue cases for one serotype should lead to decreases in number of cases for another serotype).

Our results showed the waning effects of interactions across serotypes after an independent rise of each serotype on the population level. Across serotypes, temporal effects last far longer compared to within serotype interactions, with an independent increase in cases for the serotype of interest leading to long run decreases in all other serotypes. Across-serotype interactions on the population level were found to last at least 30 weeks, with the shortest being the impact of a rise/fall in DENV-2 on DENV-1 and the longest being the impact of a rise/fall in DENV-1 on DENV-3 transmission. However, these results can also be inverted and viewed as the independent impact of a decrease in one serotype leading to a persistent rise in cases in all other serotypes. Possible explanations include waning cross-immunity on the population level to one serotype after an outbreak leading to another serotype possibly emerging as the predominant serotype and seed a new epidemic on the population level leading to multi-year epidemic cycles [4,6].

While impulse response analysis identifies the impact of an independent rise of each serotype on every other serotype, a rise in transmission of one dengue serotype may or may not be an independent event, due to other extrinsic factors such as vector population and climate. In Singapore, past studies have documented the switch between DENV-1 and DENV-2 and showed evidence that this may be a driving factor of outbreaks and may serve as a warning signal for the future occurrence of an outbreak [6,7]. Historical decomposition analysis in this study similarly revealed that a sizeable portion of reported case counts for the predominant serotypes during the 2013–2015 DENV-1, 2015–2016 DENV-2 and 2020 DENV-2/3/4 outbreaks are attributable to past interactions with other serotypes. Counterfactual analysis reveals that without these across serotype dynamics, the respective epidemics may be substantially smaller in size.

Historical decomposition analysis revealed the elevated burden of dengue due to hyperendemicity and correspondingly, dengue burden is highest in Southeast Asia—this may be partly attributable to hyperendemicity [1]. However, humans are the primary reservoir of the dengue virus [5], making it virtually impossible to remove any one serotype from active circulation. Most other regions, such as those in temperate regions and Latin America have isolated outbreaks of usually 1 and/or 2 serotypes [25]. In regions where dengue is not hyperendemic, public health officials should reduce the possibility for other serotypes to gain a foothold and enter active circulation. This can be conducted crucially with virological surveillance as well as vector control measures. Optimistically, reducing the number of serotypes in active circulation can reduce the magnitude of outbreaks for already predominant serotypes in the longer term and allow easier management and mitigation of future dengue epidemics within said regions.

Historical decompositions of past outbreaks also showed that not all case counts may be attributable to interactions across serotypes alone. The favourable equatorial climatic conditions [26,27], coupled with a large degree of urbanization [28] allow year-round vector breeding in Singapore. Paradoxically, successful vector control has also reduced the level of herd immunity in Singapore, which causes new outbreaks to occur, even as the vector breeding index remains relatively low [11]. Baseline risk factors, while subsumed in the intercept term for the VAR specification, cannot be accounted for in historical decomposition analysis and may have contributed to less than ideal approximations of the decomposition.

In terms of model building, the VAR is by nature not parsimonious due to its multivariate structure. Additional lag terms or covariates would substantially increase the number of parameters to be estimated, depending on the number of endogenous variables which are of interest. However, a major strength is that the proposed VAR framework is generalizable to many multi-strain diseases, such as influenza. One only needs to have prior biological knowledge on the direction of short term cross-immunity between different strains of the same pathogen as well as recorded case counts across time for each strain, in order to understand across strain interactions on the population level. This allows easy construction of restrictions to sign-identify a VAR. Our study primarily focused on dengue in Singapore; future work should examine whether these findings are different in other dengue settings.

## 5. Conclusion

Our work revealed the long run persistence of across-serotype dynamics and how these dynamics contribute to past outbreaks. Counterfactual analysis revealed that dengue hyperendemicity substantially elevates the severity of past outbreaks. Public health officials should aim to reduce the possibility for other dengue serotypes to gain a foothold and enter active circulation to reduce dengue burdens. Future work can also incorporate biological knowledge on the duration of cross-immunity into the sign-restriction framework to more accurately triangulate the impacts of interactions between serotypes on population level transmission.

## Data accessibility

All codes used to reproduce the study are available at https://github.com/juetaoLim/SeroSwitchSg.
